# Stroke Prevention Rehabilitation Intervention Trial of Exercise (SPRITE) - a randomised feasibility study

**DOI:** 10.1186/s12872-017-0717-9

**Published:** 2017-12-12

**Authors:** Neil Heron, Frank Kee, Jonathan Mant, Philip M. Reilly, Margaret Cupples, Mark Tully, Michael Donnelly

**Affiliations:** 1 Department of General Practice and Primary Care, Queen’s University, Belfast, UK; 2 Centre for Public Health Research, Queen’s University, Belfast, UK; 3 UKCRC Centre of Excellence for Public Health Research (NI), Belfast, Northern Ireland; 4 Primary Care Unit, Department of Public Health and Primary Care, University of Cambridge, Strangeways Research Laboratory, Cambridge, UK; 5 Patient and Public Involvement (PPI) Representative for SPRITE Studies, Belfast, Northern Ireland; 6 Department of General Practice, Queen’s University, Dunluce Health Centre, Level 4, 1 Dunluce Avenue, Belfast, BT9 7HR UK

**Keywords:** TIA, Minor stroke, Secondary cardiovascular prevention, Cardiac rehabilitation, SPRITE, ‘The Healthy Brain Rehabilitation Manual’, ‘The Heart Manual’

## Abstract

**Background:**

The value of cardiac rehabilitation (CR) after a transient ischaemic attack (TIA) or minor stroke is untested despite these conditions sharing similar pathology and risk factors to coronary heart disease. We aimed to evaluate the feasibility of conducting a trial of an adapted home-based CR programme, ‘The Healthy Brain Rehabilitation Manual’, for patients following a TIA/minor stroke, participants’ views on the intervention and, to identify the behaviour change techniques (BCTs) used.

**Methods:**

Clinicians were asked to identify patients attending the Ulster Hospital, Belfast within 4 weeks of a first TIA or minor stroke. Those who agreed to participate underwent assessments of physical fitness, cardiovascular risk, quality of life and mental health, before random allocation to: Group (1) standard/usual care; (2) rehabilitation manual or (3) manual plus pedometer. All participants received telephone support at 1 and 4 weeks, reassessment at 6 weeks and an invitation to a focus group exploring views regarding the study. Two trained review authors independently assessed the manual to identify the BCTs used.

**Results:**

Twenty-eight patients were invited to participate, with 15 (10 men, 5 women; 9 TIA, 6 minor stroke; mean age 69 years) consenting and completing the study. Mean time to enrolment from the TIA/stroke was 20.5 days. Participants completed all assessment measures except VO_2max_ testing, which all declined. The manual and telephone contact were viewed positively, as credible sources of advice. Pedometers were valued highly, particularly for goal-setting. Overall, 36 individual BCTs were used, the commonest being centred around setting goals and planning as well as social support.

**Conclusion:**

Recruitment and retention rates suggest that a trial to evaluate the effectiveness of a novel home-based CR programme, implemented within 4 weeks of a first TIA/minor stroke is feasible. The commonest BCTs used within the manual revolve around goals, planning and social support, in keeping with UK national guidelines. The findings from this feasibility work have been used to further refine the next stage of the intervention’s development, a pilot study.

**Trial registration:**

ClinicalTrials.gov Identifier: NCT02712385. This study was registered prospectively on 18/03/2016.

**Electronic supplementary material:**

The online version of this article (10.1186/s12872-017-0717-9) contains supplementary material, which is available to authorized users.

## Background

Strokes and transient ischaemic attacks (TIAs) are highly prevalent conditions [1, 2] and the 90 day risk of vascular events following a TIA or ‘minor’ stroke can be as high as 18% [3]. Therefore, the immediate period after a TIA and ‘minor’ stroke is a crucial time to intervene to reduce the risk of future strokes and the impact that these conditions have on society.

Evidence is growing regarding the contribution of change in modifiable risk factors to reductions in cardiovascular deaths [4] and there is a need to consider how to promote non-pharmacological measures within secondary prevention [5]. Despite the knowledge surrounding vascular risk factors and the recognition that TIA/‘minor’ strokes carry a significant morbidity and are often the precursors of disabling strokes, stroke remains the leading cause of adult disability [6].

Cardio- and cerebro-vascular disease share common underlying pathological mechanisms and risk factors, but cardiac rehabilitation for secondary prevention is only offered to patients in the UK with cardiovascular disease [7] and further research has been highlighted as required in assessing the impact of lifestyle interventions post-stroke and TIA [8]. The benefit of cardiac rehabilitation for secondary cardiovascular prevention is well-evidenced. Indeed a systematic review of exercise-based cardiac rehabilitation after a myocardial infarction (MI) found statistically significant reductions in re-infarction (odds ratio 0.53), cardiac mortality (odds ratio 0.64), and all-cause mortality (odds ratio 0.74) [9]. More recently, Rauch et al.’s review [10] has confirmed the benefits of cardiac rehabilitation for mortality despite recent advances in medical and surgical treatments.

Lennon et al. [11] and McKay-Lyons et al. [12] have described randomised trials of community-based cardiac rehabilitation programmes in both TIA and stroke patients. These studies did not review the use of home-based programmes, which we know are at least as effective as community-based options whilst being more cost-effective [13], and did not include a pedometer arm. These studies finished early, with no results available in their clinical trials registry entries or published. Both described a relatively long-period from the event to entry into the trial (up to 90 days for both studies). However other research has shown that vascular risk factors should be addressed as quickly as possible following the initial vascular event [14].

Prior et al. [15] have described a pilot study of a community-based cardiac rehabilitation in 100 post-TIA and ‘mild’ stroke subjects and showed important reductions in biological markers linked to cardiovascular and cerebrovascular mortality. Thus community-based cardiac rehabilitation programmes appear feasible in this patient group. However the study did not include a control group, patients were eligible for inclusion up to 1 year post-event, the researchers did not offer home-based rehabilitation and did not include pedometers in the rehabilitation programme. Other authors have also piloted community-based cardiac rehabilitation programmes in the TIA and minor stroke population, with patients eligible for inclusion up to 1 year post-event [16, 17] but no authors have previously assessed the feasibility of adapting a home-based cardiac rehabilitation programme, with or without an added pedometer intervention, for use within this patient population in the sub-acute period following diagnosis.

Compliance as well as uptake [18], particularly among women and the elderly [19], with cardiac rehabilitation programmes is an issue, which can be improved through home-based options. Cochrane Reviews have demonstrated that home-based cardiac rehabilitation programmes can result in similar health gains to hospital-based [20], or centre-based programmes [13], with home-based programmes improving adherence to the programme [21]. Moreover, home-based cardiac programmes have shown longer term sustainability of health benefits compared with hospital-based programmes [22].

Physical inactivity is one of the most important recognised risk factors for cerebrovascular disease [23] and pedometers have been shown to be effective in promoting physical activity [24] through different behaviour change methods, including goal setting, providing feedback, and monitoring of activity levels [25, 26]. Pedometers appear feasible for use by patients with stroke [27, 28] and also promote walking, which is one of the commonest forms of physical activity for older adults to engage in [29]. A systematic review on the role of exercise post-stroke has however highlighted the lack of studies of pedometers in the acute and sub-acute periods of TIA or stroke [30]. Pedometers also appear to promote physical activity in the long-term, with De Cocker et al. showing that after 4 years of follow-up, the pedometer intervention group had higher physical activity levels than the control group [31].

Comprehensive programmes, which try to alter participants’ behaviours, are complex: information about their ‘active’ ingredients, such as specific BCTs [32], would facilitate their replication and the implementation of guidelines for good clinical practice [33, 34]. Identifying behaviour change techniques (BCTs) used in different behaviour change programmes has also been identified as a national research priority [34]. Thus ‘The Healthy Brain Rehabilitation Manual’, adapted from the ‘Heart Manual’, the only validated home-based cardiac rehabilitation programme supported by the UK National Institute for Health and Clinical Excellence (NICE) for patients who have had a myocardial infarction (MI) [35], is being developed following the MRC guidelines for developing complex health service interventions [36], to maximise secondary prevention post-TIA and minor stroke.

### Research aims

The aim of this study is to assess the feasibility of evaluating the effectiveness of a novel adapted home-based cardiac rehabilitation programme, ‘The Healthy Brain Rehabilitation Manual’, with or without a pedometer intervention, initiated within 4 weeks of a first TIA or minor stroke of atherosclerotic origin. We aimed to assess rates of recruitment, completion of outcome measures and follow-up, to explore participants’ views of the programme and research methods, and to identify the BCTs used within the programme.

## Methods

### Trial registration and ethics approval

The study was approved by the Office for Research Ethics Committees, Northern Ireland (REC reference [15]/NI/0001, 21/09/2015) and registered (ClinicalTrials.gov, NCT02712385). We have followed CONSORT guidelines for reporting randomised feasibility trials [37] as well as National Institute of Health Research (NIHR) guidance for feasibility studies [38].

### Study setting and participants

Patients attending TIA/ ‘minor’ stroke assessment hospital clinics in Belfast (UK) were given information about the study by a nurse and asked for their consent to be telephoned by the lead researcher (NH) the following day to invite their participation. Those who agreed attended the Northern Ireland Clinical Research Facility (NICRF), Belfast City Hospital, for an initial meeting where, with consent, baseline data were collected.

Patients were eligible for inclusion if they were aged 18 years or older and within 4 weeks of their first symptoms of a TIA or ‘mild’ stroke. The diagnosis was made by the consultant at the clinic, based on history, neurological examination and neuroimaging [39]. Using the TOAST classification system [40, 41] only TIAs and ‘minor’ strokes attributed to atherosclerosis or small vessel occlusion were included. We excluded patients who had unstable cardiac conditions or contra-indications for exercise training [42] by screening patients using the Physical Activity Readiness Questionnaire (PAR-Q) [43]. We also excluded patients who were unable to give informed consent or had a previous cerebrovascular event.

### Data collection

At their initial meeting, lasting approximately 1 h, the researcher measured height and weight (in light clothing, using a Seca scale, model 799), waist circumference (as per [44]), resting blood pressure and heart rate (using BpTRU, model BPM-200 [45]), checked the heart rhythm manually to exclude any dysrhythmias (radial pulse for 1 min) and recorded other variables including sex, age, marital status, smoking status, alcohol intake (units in a typical week before diagnosis), time from initial event to study enrolment, level of education (high school, apprenticeship, further education college or University) and current employment. A measure of deprivation (multiple deprivation measure (MDM)) was derived from their postcode [46]. We enquired about family history of cardiovascular disease (CVD), assessed physical activity levels (validated International Physical Activity Questionnaire (IPAQ) questionnaire [47, 48]) and calculated a Mediterranean Diet Score using a validated questionnaire [49]. A 2-min walk test was performed twice, separated by a rest period of at least 30 min, and the average distance walked in metres was calculated [50]. During the 2 min of testing participants were encouraged to walk as fast and as far as they could. A Hospital Anxiety and Depression (HADs) questionnaire [51] was used to assess anxiety and depression, a EQ5D-5 L questionnaire (http://www.euroqol.org/eq-5d-products/eq-5d-5l.html) to assess quality of life, a Modified Rankin scale [52] to assess level of disability and a Prochaska stages of change questionnaire relating to physical activity was administered [53]. All participants were offered VO_2max_ exercise testing via either a treadmill or bike.

### The intervention – ‘The Healthy Brain Rehabilitation Manual’

The development of a novel home-based rehabilitation programme for a TIA/‘minor’ stroke population followed the Medical Research Council (MRC) guidelines for developing complex health service interventions [36, 54]. In terms of programme content, ‘The Healthy Brain Rehabilitation Manual’ contained an introduction, telling the user how to use the manual, medical and social information about TIAs/‘minor’ strokes and how to set goals and action plans for changing certain aspects of their lives. There was then sections focusing on topics relevant to cardiovascular risk (smoking, physical and sexual activity, mental health issues (primarily anxiety and depression), community resources (e.g. smoking cessation support; exercise classes), diet and secondary prevention medication). The manual was supported with telephone follow-up by a health professional, a General Practitioner (GP). Participants were given advice about how to know that they were participating in moderate intensity activity. Those with a pedometer were advised that a cadence of 100 steps/min corresponds to moderate physical activity and for those who did not have a pedometer, they could use the ‘talk/sing test’ [55]. This was explained to patients at their baseline assessments and was included in the manual as well as being reinforced through the telephone follow-up.

Two trained review authors (NH, MAT) independently assessed the manual to identify BCTs included, using Michie’s BCT taxonomy [32] of 93 hierarchically clustered techniques and a narrative approach was used to describe the use of BCTs within the rehabilitation programme. They met to discuss the BCTs which they had identified and resolve any discrepancies. A third reviewer (FK) was available to arbitrate in case consensus could not be reached, but was not required.

### Randomisation and blinding

Computer generated randomisation was carried out prior to recruitment and the allocations were concealed in sealed, opaque envelopes until baseline assessments were completed. Post-intervention assessments were undertaken by NH, who was not blinded to intervention allocation.

### Study design

There were 3 study arms: the control group, Group 1, received current standard post-TIA/minor stroke care as per current UK guidelines [35, 56]. In addition to standard care, Groups 2 and 3 received the intervention programme (‘The Healthy Brain Rehabilitation Manual’). Group 3 also received a pedometer or a Fitbit Charge, with each participant choosing which they wanted to use, and being encouraged to keep a daily step-count diary. NH advised Groups 2 and 3 regarding the use of the manual and pedometer/diary at the end of their initial meeting and assessment. Participants in Groups 2 and 3 were informed about the national UK physical activity guidelines as well as how to achieve moderate and vigorous physical activity intensity [57]. The pedometer was used to allow participants to set and monitor goals to increase their physical activity levels.

All participants, including Group 1, were telephoned at 1 and 4 weeks to answer any questions regarding their care or use of the manual and, for Group 3, NH encouraged participants to self-set step count targets after reviewing the previous week’s daily step counts [24]. Step counts were recorded, as reported by participants at the end of week 1 and diary records were reviewed by NH at the 6-week follow-up. Average step-counts for weeks 1 and 6 were calculated by adding the daily totals and dividing by the number of days/week worn by the participant. During the initial meeting and telephone contacts NH used motivational interviewing techniques [58], guided by the theory of planned behaviour [59] and adopting the ‘5 As’ approach to behaviour change [58], which have all been utilised within different healthcare settings [60], including primary care and the community.

### Data treatment and statistical analysis

No formal power calculation was undertaken as this was a feasibility study but 5 patients in each of 3 treatment groups was considered sufficient to allow assessment of the feasibility of recruitment, conduct of proposed assessments and retention. It was also considered that 15 patients’ views could provide useful information regarding our research methods and the acceptability of the intervention programme and its refinement for potential use in a pilot trial. Descriptive statistics were reported for baseline and post-intervention measurements, using Statistical Package for Social Sciences (SPSS, version 23) but the main outcomes were rates of recruitment, retention and completion of measures and the acceptability of the intervention.

### Qualitative work

All participants were invited to attend a focus group which took place at least 2 months after their completion of the study and they were encouraged to bring their partner or a family member to the focus group. The primary questions of the topic guide (Appendix) related to research participation and the acceptability of the different stages of the research study [61]. The focus group discussion was led by NH, audio-recorded with the consent of participants, lasted approximately 1 h and was transcribed by NH. Content analysis was undertaken with the practical purpose of eliciting views about the acceptability and usability of the intervention and research methods and how these could be refined. NH, a male GP, and MD, a male health services researcher/health psychologist, read and reread the transcripts and coded the content independently. NH and MD met to discuss the main areas covered within the transcripts. MC, a female professor of GP, acted as a referee as required as well as appraising critically the categories and the degree to which the transcript extracts and quotations supported the themes. The transcripts were not returned to participants for comment and/or correction and participants were not asked to provide feedback on the qualitative results. NH has basic training in qualitative research methods whilst MD and MC are experienced qualitative researchers. The independent results of the qualitative analysis and data interpretation were discussed with the entire research team, to ensure clear definition of themes and that appropriate supporting evidence was identified for each. The reporting of the qualitative study and findings followed the guidance set out in the Consolidated Criteria for Reporting Qualitative Research (COREQ) checklist [62].

## Results

### Recruitment, retention and completion of assessment measures

During an 18-week recruitment period (March to July, 2016) 107 patients with confirmed TIA/minor stroke attended the hospital TIA clinic. From the hospital data recorded, we were unable to determine how many of these were eligible for the study but 28 eligible patients (15 male; 13 female) agreed to telephone contact from NH. Of these, 15 (10 male; 5 female; 53.6%) consented to participate. All of these completed the study and attended for 6-week follow up assessment. Participants completed all the outcome measures apart from the maximal exercise tests for VO_2max_ assessment, which was declined by all participants at baseline and follow-up. Four participants in Group 3chose to use a Fitbit Charge initially as their pedometer; 2 reported positive experiences using this device, particularly in goal setting and competition with other users but 2 others were unable to use it and transferred to use a pedometer, Yamax Digi-Walker CW-701, with which no patients reported any problems.

### Baseline characteristics

The participants’ mean age was 69 years; 9 were diagnosed with a TIA and 6 a minor stroke (Table 1). Mean time from event to enrolment was 20.5 days. Only one had attained University level education; most had retired. The majority [10] lived in the 50% least disadvantaged areas of Northern Ireland. Although 7 were ex-smokers, only one participant currently smoked; mean alcohol intake was <14 units/week. Ten participants had a first degree relative with CVD and most participants were married (13/15). Baseline IPAQ scores indicated that 10 were either inactive or minimally active and many (9/15) reported sitting for over 5 h daily. In their first week, Group 3 participants averaged over 8000 steps/day. Baseline mean systolic and diastolic blood pressure (SBP and DBP) were <140/90 mmHg in all groups. The total HADs score was elevated in all 3 groups, particularly for anxiety symptoms. Most participants within the study were classed as ‘overweight’ as per their BMI.

**Table 1:** Baseline characteristics of participants

	Group 1 (Control)	Group 2 (Manual)	Group 3 (Manual + pedometer)
Number of patients	5	5	5
Sex (M – male, F – female)	4 M 1F	4 M 1F	2 M 3F
Diagnosis	1 TIA	4 TIA	4 TIA
	4 Minor stroke	1 Minor stroke	1 Minor stroke
Mean age (years)	76.2	67.8	63
Mean time (days) event to enrolment (Standard Deviation (SD))	19.8 (SD 7.09)	22.2 (SD 9.18)	19.6 (3.58)
Level of education	3 High school	3 High school	1 High school
	2 Further Education College	2 Further Education College	3 Further Education College
			1 University
Employment	1 Employed	2 Employed	1 Employed
	4 Retired	1 Unemployed	4 Retired
		2 Retired	
Multiple deprivation measure (MDM) median (range)^a^	784 (51–863)	413 (250–726)	681 (333–825)
Family history of cardiovascular disease (<55 years for males, <65 years old for females)	4	3	3
Marital status	1 Single	5 Married	4 Married
	4 Married		1 Divorced
Smoking status	2 non-smokers	2 non-smokers	3 non-smokers
	3 ex-smokers	2 ex-smokers	2 ex-smokers
		1 current smoker	
Modified Rankin scale			
0	2	2	4
1	1	1	1
2	1	1	0
3	0	1	0
4	1	0	0

^a^Multiple deprivation measure (MDM) is calculated from the subject’s postcode and is a marker of spatial deprivation

### Post-intervention assessment

Groups 3’s mean daily step counts increased over the 6 weeks of the intervention by 1407, with a concomitant fall in the numbers in the IPAQ categories of ‘inactive’ and ‘minimally active’ (Table 2). Participants reported good compliance with the pedometer, wearing it on most days, as recorded in the step-count diary. IPAQ data for Group 2 also showed an increase in physical activity and a reduction in hours sitting per day. The two-minute walk distance increased in all groups, with the greatest increase in Group 3. HADs scores, particularly for anxiety, improved in Groups 2 and 3. There was a wide variation in the rest of the measurements and overall they showed only small changes.

**Table 2:** Study Group Baseline and Post-Intervention Measurements (Mean (Standard Deviation (SD)) and categorical values)

	Group 1 (Control) – baseline	Group 1 – post-intervention	Group 2 (Manual) – baseline	Group 2 – post-intervention	Group 3 (Manual + pedometer) – baseline	Group 3 – post-intervention
IPAQ category						
Inactive	2	1	3	1	1	0
Minimally active	1	4	1	0	2	1
Health-enhancing physical activity levels	2	0	1	4	2	4
IPAQ continuous score (Mean (SD))	1514.6 (SD 1470.1)	1093.8 (SD 851.1)	870.4 (SD 948.2)	4366.2 (SD 3140.8)	1531.2 (SD 902.5)	5335.8 (SD 3133.6)
IPAQ number of hours sitting/day	6.20 (SD 4.10)	6.3 (SD 4.0)	4.80 (SD 1.5)	3.2 (SD 1.3)	5.00 (SD 1.0)	3.8 (SD 1.3)
Steps/day					8356	9762.8 (SD 3473.5)
Stages of change for physical activity						
1	1	1	1	0	0	0
2	0	0	3	0	1	0
3	2	2	0	1	1	0
4	0	0	0	3	2	4
5	2	2	1	1	1	1
Mediterranean diet total score	5.6 (SD 3.1)	5.8 (SD 2.5)	6.2 (SD 3.3)	8.4 (SD 2.3)	6.0 (SD 2.2)	8.0 (SD 1.4)
Number of pieces of vegetables/day	1.6 (SD 1.1)	1.8 (SD 1.5)	2.2 (SD 1.8)	2.4 (SD 0.6)	2.0 (SD 1.2)	2.8 (SD 0.8)
Number of pieces of fruit/day	2.2 (SD 1.9)	2.6 (SD 1.5)	2.2 (SD 1.3)	2.8 (SD 1.3)	3.4 (SD 0.9)	4.0 (SD 1.4)
Systolic blood pressure (mmHg)	135.8 (SD 8.4)	135.8 (SD 24.8)	136.2 (SD 15.2)	128 (SD 2.0)	129.4 (SD 15.2)	131.0 (SD 12.9)
Diastolic blood pressure (mmHg)	76.4 (SD 10.8)	76.8 (SD 10.0)	82.8 (SD 13.9)	74.8 (SD 4.0)	77.2 (SD 8.1)	80.4 (11.2)
Resting heart rate (beats per minute)	66.2 (SD 3.0)	71.6 (SD 8.4)	65.6 (SD 0.9)	74.8 (SD 4.0)	75.0 (SD 9.4)	73.8 (SD 10.8)
2 min walk test performance (metres walked)	109.5 (SD 46.3)	118.2 (SD 28.2)	128.8 (SD 29.1)	149.8 (SD 28.7)	136.5 (SD 41.5)	163.4 (SD 23.4)
Weekly alcohol intake (units/week)	6.4 (SD 12.1)	5.6 (SD 8.4)	5.6 (SD 10.4)	1.6 (SD 2.6)	2.8 (SD 3.0)	2.4 (SD 2.6)
HADs total score	10.6 (SD 6.5)	11.0 (SD 5.2)	10.4 (SD 4.7)	7.4 (SD 5.5)	7.00 (SD 4.2)	3.8 (SD 2.7)
HADs Anxiety score	6.0 (SD 4.1)	6.2 (SD 3.6)	6.0 (SD 2.1)	4.2 (SD 3.5)	5.2 (SD 2.8)	3.0 (SD 2.0)
HADs Depression score	4.6 (SD 3.2)	4.8 (SD 2.4)	4.4 (SD 3.1)	3.2 (SD 2.4)	1.8 (SD 1.9)	0.8 (SD 0.1)
EQ5D5L overall score	0.7 (SD 0.4)	0.7 (SD 0.4)	0.9 (SD 0.1)	1.0 (SD 0.1)	1.0 (SD 0.0)	1.0 (SD 0.1)
EQ5D5L VAS score	66.0 (SD 32.1)	53 (SD 29.7)	72.0 (SD 17.5)	86 (SD 6.5)	83.0 (SD 18.2)	85.8 (SD 17.6)
Weight (kg)	80.6 (SD 10.9)	80.3 (SD10.9)	80.7 (SD 8.1)	80.1 (SD 8.2)	79.6 (SD 15.6)	79.4 (SD 16.5)
BMI (kg/m^2^)	27.3 (SD 2.5)	27.2 (SD 2.5)	29.7 (SD 3.2)	29.5 (SD 3.4)	28.2 (SD 2.2)	28.1 (SD 2.2)
Waist circumference (cm)	97.7 (SD 5.7)	97.7 (SD 5.7)	99.9 (SD 6.1)	98.7 (SD 6.5)	101.2 (SD 11.3)	100.4 (SD 11.1)

### Qualitative findings

Seven research participants (3 male; 4 female) and one partner (female) attended the focus group. Their ages ranged from 55 to 82 years. Four participants were from Group 3, 2 from Group 1 and 1 from Group 2. Qualitative findings were analysed in order to determine participants’ views of the manual, the study design and changes needed for a pilot study of the effectiveness of a novel home-based programme for rehabilitation for patients with TIA or minor stroke. The analysis, supported by anonymised quotes (coded by age (years) and sex (M/F)), is reported within three themes relating to the content of the data.

#### Use of the manual

All participants had positive views about the manual. Its content provided reassurance and support, particularly in differentiating between symptoms for which they should seek medical help and those that were not of such significance. Some reported how they referred to it after having received varying information about their condition and risk factors from different healthcare professionals.

“…there was some days when I was panicking a bit and I got the book out and I was like, no, that symptom is ok, that’s normal….” (F)

“I think this (the manual) should be at doctor (GP) surgeries as well because when you go to speak to the doctor you get conflicting advice at times.” (F)

Participants’ comments reflected their fear and uncertainty about their future health, attributed to their experience of sudden onset symptoms of their TIA or minor stroke. Some appeared to be in denial of their diagnosis, based on a rationale that their symptoms had been mild (predominantly affecting vision and speech; one had right-sided weakness) and transient but their comments also reflected a sense of uncertainty. However, the manual was welcomed by all as a reference source for credible information that helped them to understand their diagnosis.

“It (the TIA) just frightened me and knocked my confidence… because you get no warning.” (F)

“You know, for just 10 minutes’ worth of symptoms, surely nothing serious could have happened?” (F)

“I thought it was excellent…….. like a bible” (M)

Family members also used the manual in supporting decision-making about seeking medical help. Some did so effectively but others were less helpful: one participant’s comments indicated how her family members wished they could deny the significance of her symptoms.

“…I was just going to go to my bed but my daughter phoned and I said to her the symptoms I was having and she took me straight to Accident and Emergency (A&E) department.” (F)

“My daughters didn’t want to read it because if you read it, then it’s true…” (F)

Other information in the manual provided reassurance for those who felt guilty about not being able to fulfil their previous work-life commitments, particularly as they had no visible physical manifestations of illness. Information regarding the relevance of healthy lifestyle behaviours in helping to reduce risk of further events was valued and participants reported having continued to access it after the study programme had ended: some reported that they put the manual in a prominent place to remind them to sustain preventive behaviours. Other comments indicated how family members used the manual to encourage maintenance of healthy behaviours. Those who had received positive feedback about progress in reducing their risk factors attributed this to having followed guidance in the manual.

“I felt an awful fraud cause I was off work but there was nothing physically wrong with me…nothing to show ” (F)

“I lift it every morning, read a wee bit, remind myself why I’m not smoking, why I’m not eating a whole load of pastries and why I’m avoiding salt….I might just read a line but it’s the very fact that it’s sitting there, reminding me of what to do right….I find that very important…” (M)

“my grand-daughter has read it from page to page and every time she comes up to see me, she’s like, ‘grandad, have you done that? Are you keeping to that?” (M)

“The fact that my blood pressure is a lot lower is also very encouraging for me to stay with the programme as I feel the things which I have done have definitely helped me…” (F).

#### The study design

##### Recruitment and randomisation

None of the participants considered that any change was needed to the process of recruitment or method of being allocated to study groups.

##### Intervention components – telephone follow-up

The structure and timing of telephone follow-up calls were well received by all participants who considered that they reduced their need to seek other medical advice. Comments revealed how participants valued the opportunity to share concerns with a professional and ask questions. For example, many participants expressed how they had fears for the implications of their diagnosis on future travel plans and being able to obtain travel insurance

“………they made me feel that there was someone out there interested in me and who cares for me.” (M)

“...it put your mind at rest because you were thinking, NH is phoning me soon, so I don’t need to go to see the GP. I enjoyed the explanations.” (F)

“…..are we going to get to go on holiday and will I be able to fly?” (F)

Intervention components - pedometers

All Group 3 participants enjoyed using their pedometers, setting step-count targets and competing with others regarding their achievement of these. One participant reported her appreciation of the Fitbit with its provision of weekly email feedback on her performance. However, two participants discontinued using the Fitbit despite research team support, one because of battery problems and one lacked confidence in its accuracy: both used the Yamax pedometer without difficulty.

“…it’s a competition between me and the wife who walks the furthest” (M)

“ It was just so addictive …. I had that visual target to aim for…It would also send me an email at the end of the week, telling me how much activity I had done and I just thought it was brilliant.” (F)

“…and the pedometer would always be less (in step-count measurement compared to Fitbit)….” (F)

Outcome assessments

All participants were also content with the number and duration of assessments and with all outcome measures except treadmill exercise testing. They were apprehensive that they would be unable to complete it, given their recent diagnosis. However, 4 participants expressed a readiness to consider undertaking it at the time of the focus group, approximately 2 months after having completed the rehabilitation programme.

“I doubt that I would have been able to do the treadmill exercise test within 4 weeks of having the TIA….” (M)

“I would be interested in doing it now…” (Two Fs)

#### Suggested changes

The only changes suggested by study participants related to the manual. One suggestion was to move the explanations about TIA and minor stroke to the beginning of the manual and another was to include a patient’s story.

“…. the explanation for the TIA (and minor stroke) is at the back of the manual. I think it should be at the start?’” (F)

“people are interesting and it’s good to hear their experiences…” (F)

### BCTs used within the manual

Overall, 36 individual BCTs, from 14 different BCT groups were utilised. Examples of how the BCTs were used in each section of the manual are included in Additional file 1: Tables S1 to S8. Within Section 1 (Smoking) 16 individual BCTs were used and in section 2, dealing with physical/sexual activity, with 11 being used in total. The commonest BCTs used were “1.1 Goal setting (behaviour)”, “3.1 Social Support (unspecified)” and “3.3 Social support (emotional)” – all being used within 5 of the 8 manual sections. The commonest groups of BCTs used within the manual were “1 (Goals and Planning)”, being detected 18 times and Social Support (12 times). Two groups of BCTs were not used within the manual, “14 (Scheduled consequences)” and “16 (Covert learning)”.

## Discussion

**Fig. 1:**
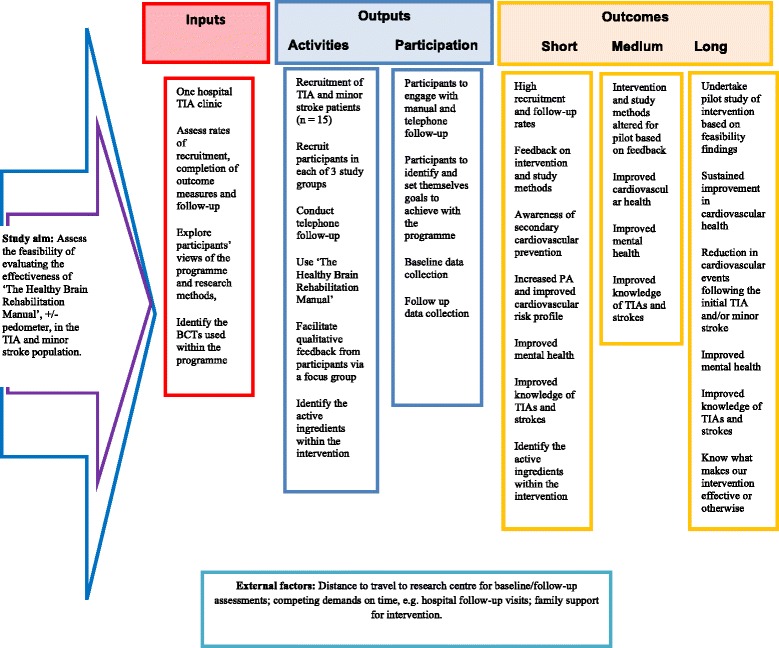
Intervention logic model

These findings indicate that the evaluation of a novel home-based rehabilitation programme, ‘The Healthy Brain Rehabilitation Manual’, implemented within 4 weeks of a first TIA or ‘minor’ stroke is feasible. There was 100% retention of participants and more than 50% of patients who were invited by the researcher agreed to participate, although we could not be certain that all eligible patients consented to allow contact by the researcher. All but one of the proposed assessments, the VO_2max_ testing, were fully completed at baseline and follow-up. This study also illustrates the acceptability of pedometers as an appropriate method of promoting physical activity to generally inactive TIA and minor stroke patients. The rehabilitation programme was centred on the use of ‘goals and planning’ and social support as BCTs as well as the manual being a credible source of information to promote behaviour change. A Logic Model has been developed for this feasibility study and is included in Fig. 1 [63, 64].

### Comparison with previous literature

In comparison with previous studies of community-based cardiac rehabilitation for patients with TIA or ‘mild’ stroke, we have achieved higher rates of recruitment (50% of those invited) and retention (100%). A feasibility study [17] reported 62% retention of 85 patients enrolled and, in a pilot study [15], approximately 50% of 100 invited patients consented to participate and roughly 80% completed the study. Of note, home-based cardiac rehabilitation programmes are reported to improve programme adherence [21] and to show longer-term sustainability of health benefits compared with hospital-based programmes [22].

An interesting finding was that all participants declined to undertake a maximal VO_2_ test, both pre- and post-intervention, whereas all other assessments were well received. To our knowledge, this is the first study to report this finding. Exercise testing is safe to undertake among patients with TIA and stroke [65] as well as generally within the elderly population [66] but our participants felt unable to complete it. Thus, for further work, we have omitted the maximal exercise test, adding the Timed Up and Go test [67] and accelerometer assessments of physical activity pre- and post-intervention, which are reliable objective measures of physical activity in stroke survivors [68] and amongst the elderly population [69]. However, the Timed Up and Go test is a short test of physical performance which can be insufficient to capture deficiencies in cardiovascular endurance. Thus, we plan also to use accelerometry to assess participants’ physical activity levels, as well as to measure gait speed during the 2-min walk tests as this has been shown to be an important predictor of morbidity and mortality in the elderly population [70].

The Group 3 participants increased their physical activity by 1400 steps/day over the study period of 6 weeks. Indeed, walking ability has been demonstrated to be strongly associated with cardiorespiratory fitness [71] as well as being helpful in guiding prognosis in patients with cardiovascular disease [72]. Moreover, an increase in steps/day walked after cardiac rehabilitation has been shown to reduce overall risk of mortality and hospitalization [72] Therefore the improvement in physical activity levels (steps/day) demonstrated within this feasibility study could contribute to making our TIA and ‘minor’ stroke patients live longer as well as healthier lives. Previous studies have also shown that there is potential for larger increases to be achieved with pedometer interventions [26, 73–75], with potential to reduce cardiovascular risk factors further, particularly amongst those who are the least active. It has been suggested that healthcare workers should enquire regularly about the walking status of their patients and that this should be viewed as an another ‘vital sign’ in healthcare [76]. Pedometers are also accurate and reliable in measuring ambulatory activity [75, 77–79] whilst also being relatively inexpensive. Yamax pedometers have been shown to be the most accurate waist-borne instrument [75, 77].

The intervention appeared to improve quality of life as measured by the EQ-5D-5 L VAS scale and mental health, as measured by the HADs questionnaire [51], although numbers were small and no significance testing was carried out. In keeping with previous work [80] which found a prevalence within TIA survivors of anxiety symptoms of up to 30% and depression symptoms of up to 21%, our focus group participants were anxious and fearful about having future events. Patients appreciated the explanations and reassurance provided by the manual.

It is important for complex health service interventions to be evidence-based and to know that they are not only effective but how and why they are effective [81]. Specifying and describing how a programme of behaviour change actually works is also advocated by UK national guidelines [34], with journal editors requesting detailed descriptions of the active intervention within their reporting guidelines [82]. Thus, the active ingredients within a programme should be identified [83]. NICE [34] have advocated the use of BCTs which have been proven to be effective in promoting behaviour change, particularly ‘goals and planning’; ‘feedback and monitoring’; and, ‘social support’. ‘The Healthy Brain Rehabilitation Manual’ includes goal-setting for behaviour change, with agreed action plans and relapse prevention (‘if-then’) plans for each behavioural cardiovascular risk factor. These goals and plans are reviewed and refined in follow-up contacts with the health professional/facilitator. The manual includes feedback and monitoring as a BCT, for example, based on review of pedometer step counts. Social support is promoted through contact with health professionals and encouraging the person to share the manual with their family and friends and get them to join in the behaviour change.

### Strengths and limitations

This is a feasibility study and therefore no statistical analysis was undertaken on the outcomes. Baseline assessments were completed before participants were allocated to different groups, to avoid allocation bias. The qualitative work undertaken included participants from each treatment group, of varying age and both sexes, with a range of different symptoms and experiences and they identified valued components of the rehabilitation programme. The educational attainment of our participants was less than third level (post secondary school education), with only one having a University degree and no one reported any difficulty in following guidance or understanding information within the manual. This was reassuring since, in developing it, we had performed a readability check (http://www.webpagefx.com/tools/read-able/check.php), showing it to be readily understandable to 13 to 14 year olds.

The pragmatic approach, whereby TIA and ‘minor’ stroke diagnosis was made by the lead clinician at each clinic, may have led to variation in the case mix due to differing interpretation of clinical data but this was accepted as a reflection of ‘real-world’ practice. There was no post-intervention blinding of assessments so that it is possible that some measurement bias may have occurred. Also, Group 3 participants were not blinded to their step counts in the first week of the study, so that the baseline measure may be inflated and not a true reflection of levels of physical activity at this time in TIA and minor stroke patients.

Whilst initial discussions with clinical staff regarding the process of identification and invitation of eligible patients had included a plan to record anonymously the numbers of all eligible patients, this information was not recorded. Thus, although data suggested that 79 of the 107 clinic attendees during the recruitment period were ineligible, this could not be confirmed and limits our interpretation of the feasibility and acceptability of the research.

A strength of our study is the careful approach we have taken to identifying the ‘active ingredients’ of the intervention. However, we recognise that our focus was on the manual content and that other BCTs were also involved in the delivery of the programme, during telephone contacts. No monitoring of the fidelity of these contacts was undertaken but this could be achieved in further study.

### Implications for future work

In planning further development and evaluation of the intervention, ‘The Healthy Brain Rehabilitation Manual’ we will conduct a pilot study, involving other hospital clinics and ensuring that information is recorded regarding all eligible patients. In setting step-count goals for physical activity, only Yamax Digi-Walker CW-701 pedometers will be used. We will discard the maximal VO_2max_ exercise test and instead use the Timed Up and Go test [67] pre- and post-intervention, with accelerometer assessments, which provide reliable objective measures of physical activity in stroke survivors [68] and older people [69]. We will also include a ‘patient story’ in the ‘The Healthy Brain Rehabilitation Manual’, in-keeping with previous authors’ work [84], and will place information about TIAs and strokes at the start of the manual.

## Conclusion

Our findings show the feasibility, acceptability and potential significance of implementing, early after TIA or ‘minor’ stroke, a novel home-based programme, ‘The Healthy Brain Rehabilitation Manual’, with or without an added pedometer. The main BCTs utilised within the manual included use of a credible source, social support and goal setting, in keeping with current UK national guidance for behaviour change. This preliminary work has informed the design of a pilot study which is in progress, with longer follow-up, recruitment from a range of settings and refined methodology (clinicaltrials.gov (NCT02712385)). This future work should provide evidence of the value of early intervention that is focused on behaviour change for patients following TIA or minor stroke.

## Appendix

### Focus group topic guide

**Impact of the diagnosis/illness on the patient:**

1. After the diagnosis, what was your biggest problem, e.g. fatigue?
2. How did the diagnosis affect your life?

**Views on the manual:**

3) What do you think of the manual?
4) What do you think about the general level of information included within the booklet – too much/too little?
5) What do you think of the length of the booklet? Is it too short? Is it too long?
6) What do you think of the layout of the booklet? Does it need more colour, pictures, different font size, etc.?
7) Would you like a patient story within the book – detailing their thoughts/emotions after being diagnosed with a TIA/minor stroke?
8) Are there any risk factors which haven’t been covered, which you would like included? Are there any risk factors on which you would like more information to be given – or less?
9) Did family members read the manual? What did they think? Anything which you particularly enjoyed about the manual?
10) Anything you would improve about the manual?

**Exercise programme within the manual:**

11) Is there anything in particular which would worry you about exercising or asking people to exercise after a TIA/stroke?
12) What do you think of the exercise programme? Would you be able to do this within your own home? Is it hard enough?

**Study duration and future goals:**

13) What goals/aims are important to you now that you have been diagnosed and are on the path to recovery?
14) What did you think about the duration of the study follow-up? Longer/shorter?

**Review of FitBit v pedometer:**

15) What are your views on the pedometer?
16) How did people find using the FitBit compared to the pedometer? What did you prefer/ why?
17) What made you stop using the FitBit?

**Research assessments and exercise treadmill test:**

18) What did people think about how they were invited and then recruited into the research study?
19) What did people think about the research assessments?
20) What did people think about the amount of time involved in the research assessments?
21) Why did people refuse to consent to the treadmill/bicycle exercise test?

## Additional file

Additional file 1: Table S1. BCTs utilised within the Manual Preface. BCTs used within the Manual’s preface. **Table S2.** BCTs utilised within the Manual Introduction. BCTs used within the Manual’s Introduction. **Table S3.** BCTs utilised within Section 1 – Smoking. BCTs used within section 1 of the manual. **Table S4.** BCTs utilised within Section 2 – Physical Activity. BCTs used within section 2 of the manual. **Table S5.** BCTs utilised within Section 3 – Healthy Eating and Alcohol. BCTs used within section 3 of the manual. **Table S6.** BCTs utilised within Section 4 – Stress and Fatigue. BCTs used within section 4 of the manual. **Table S7.** BCTs utilised within Section 5 - Medication. BCTs used within section 5 of the manual. **Table S8.** BCTs utilised within Section 6 – Community Support. BCTs used within section 6 of the manual. (DOC 197 kb)

## Abbreviations

A&E: Accident and Emergency
COREQ: Consolidated Criteria for Reporting Qualitative Research checklist
CR: Cardiac rehabilitation
DBP: Diastolic blood pressure
HADS: Hospital Anxiety and Depression score
IPAQ: International Physical Activity Questionnaire
MI: Myocardial infarction
MRC: Medical Research Council
NICE: National Institute of Health and Care Excellence
NICRF: Northern Ireland Clinical Research Facility
PPI: Patient and public involvement
SBP: Systolic blood pressure
SD: Standard deviation
SPRITE: Stroke Prevention Rehabilitation Intervention Trial of Exercise
TIA: Transient ischaemic attack
WHO: World Health Organisation
