# Supplementary material for: Stroke Prevention Rehabilitation Intervention Trial of Exercise (SPRITE) - a randomised feasibility study
Source: BMC Cardiovasc Disord. 2017 Dec 12;17:290. doi: 10.1186/s12872-017-0717-9 (PMC5727948; doi:10.1186/s12872-017-0717-9)
Supplement: Additional file 1: Table S1. — BCTs utilised within the Manual Preface. BCTs used within the Manual’s preface. Table S2. BCTs utilised within the Manual Introduction. BCTs used within the Manual’s Introduction. Table S3. BCTs utilised within Section 1 – Smoking. BCTs used within section 1 of the manual. Table S4. BCTs utilised within Section 2 – Physical Activity. BCTs used within section 2 of the manual. Table S5. BCTs utilised within Section 3 – Healthy Eating and Alcohol. BCTs used within section 3 of the manual. Table S6. BCTs utilised within Section 4 – Stress and Fatigue. BCTs used within section 4 of the manual. Table S7. BCTs utilised within Section 5 - Medication. BCTs used within section 5 of the manual. Table S8. BCTs utilised within Section 6 – Community Support. BCTs used within section 6 of the manual. (DOC 197 kb) [file 12872_2017_717_MOESM1_ESM.doc]

**Table S1** – BCTs utilised within the Manual Preface

| **BCT Label** | **BCT group** | **Example of how the BCT was used** |
| --- | --- | --- |
| 2.4 Self-monitoring of outcome(s) of behaviour | 2. Feedback and monitoring | “Please remember to visit them (GP/practice nurses) regularly for health advice, e.g. to monitor your BP…” |
| 3.1 Social support (unspecified) | 3. Social support | “This manual can also be shared with your family and friends…as well as with your General Practitioner (GP) and practice nurse” |
| 3.2 Social support (practical) | 3. Social support | “The facilitator, who will contact you by telephone, will help you go through the book.” |
| 5.3 Information about social and environmental consequences | 5. Natural consequences | “….giving you information about TIAs, minor strokes, your brain and the impact which your diagnosis can have on your life.” |
| 9.1 Credible source | 9. Comparison of outcomes | “Your facilitator, GP and practice nurse are good sources of health information.” |
| 13.2 Framing/Reframing | 13. Identity | “This manual contains information about ways you can help yourself to feel good now and remain healthy in the future.” |

**Table S2** – BCTs utilised within the Manual Introduction

| **BCT Label** | **BCT group** | **Example of how the BCT was used** |
| --- | --- | --- |
| 1.1 Goal setting (behaviour) | 1. Goals and planning | “Have realistic goals.”  Patient encouraged to keep a goal and action plans diary and write this down within the manual. |
| 1.3 Goal setting (outcome) | 1. Goals and planning | “Goal – stress (reduction). Plan – I will join a yoga class. I will find out about where there are yoga classes happening and I will sign up to attend a class once a week starting from next week.” |
| 1.4 Action planning | 1. Goals and planning | “Make an action plan.”  Patient encouraged to keep a goal and action plans diary and write this down within the manual. |
| 1.7 Review outcome goal(s) | 1. Goals and planning | “Write a few goals and action plans down for you to try to achieve over the next few weeks. You can discuss how to achieve these with your facilitator or other health professional.” |
| 1.8 Behavioural contract | 1. Goals and planning | The goals and action plans diary is agreed with the facilitator. |
| 3.1 Social support (unspecified) | 3. Social support | Advice provided about what to do if they suspect they are having another TIA or stroke. |
| 3.2 Social support (practical) | 3. Social support | “….try to contact someone who could come and be with you while you are waiting for the ambulance.” |
| 5.1 Information about health consequences | 5. Natural consequences | “The fact that you have had a TIA or minor stroke doesn’t mean that your brain is worn out or that you are ‘finished’ but should serve as a warning to you that you need to improve your health.”  “Changing the way you live, such as making small changes to your diet or getting more exercise, can reduce your risk of having another transient ischaemic attack (TIA) or stroke….” |
| 5.6 Information about emotional consequences | 5. Natural consequences | “These changes can also make you feel better and more energetic.” |
| 11.2 Reduce negative emotions | 11. Regulation | “After a TIA or stroke it is common to notice unusual feelings in your body much more than you did before. It is important not to get too worried about this. Worrying and thinking about these sensations can often make them seem worse.” |
| 13.2 Framing/ reframing | 13. Identity | “The good news is that it’s well on the way to recovery already.” |
| 15.1 Verbal persuasion about capability | 15. Self-belief | “Things to remember:   - The brain has miraculous powers of recovery. - It is one of the strongest and most adaptable organs in your body. - It is also capable of doing much more work than most of us ever need.”   “People who have realistic goals and plan changes have greater success in changing the way they live.” |

**Table S3 – BCTs utilised** within Section 1 - Smoking

| **BCT Label** | **BCT group** | **Example of how the BCT was used** |
| --- | --- | --- |
| 1.1 Goal setting (behaviour) | 1. Goals and planning | “Weigh these up for yourself and decide whether you are ready to quit.” |
| 1.2 Problem solving | 1. Goals and planning | “Identify situations that will be difficult and plan how you’ll cope…” |
| 1.4 Action planning | 1. Goals and planning | “Try taking chewing gum or something healthy rather than a cigarette.”  “Some people say that smoking is good for them because it helps them to relax. If this is the case for you, think of other ways to relax. Perhaps going for a walk, instead of having a cigarette, might make you feel good?” |
| 3.1 Social support (unspecified) | 3. Social support | “Perhaps a friend or someone in your family wants to stop too?”  “For those experiencing specific difficulties and challenges, there is specific help available in the community and your facilitator can help identify this.” |
| 4.1 Instruction on how to perform the behaviour | 4. Shaping knowledge | “If you enjoy a cigarette after a meal, clean your teeth after eating if possible.” |
| 4.2 Information about Antecedents | 4. Shaping knowledge | “Stick with it…..   - even though you may have withdrawal symptoms once you stop smoking. These should disappear within a week or two. - Don’t give in to cravings – these usually only last a few minutes and then pass.” |
| 5.1 Information about health consequences | 5. Natural consequences | “It is never too late to stop smoking and the benefits begin as soon as you give up:   - Within days, your blood is less likely to clot. - Within 5 years, the risk of a heart attack falls to half that of a smoker.   Within 10 years, you will have about the same risk of heart and brain disease as someone who has never smoked.” |
| 8.2 Behaviour substitution | 8. Repetition and substitution | “If you normally have a cigarette first thing in the morning, get up and have a shower instead.” |
| 8.4 Habit reversal | 8. Repetition and substitution | “If you like a cigarette with tea or coffee, try changing your drink to water or a fruit juice.” |
| 9.2 Pros and cons | 9. Comparison of outcomes | “First, think about the reasons you like smoking and the reasons you would like to quit smoking.” |
| 10.3 Non-specific reward  10.6 Non-specific incentive  10.7 Self-incentive  10.9 Self-reward | 10. Reward and threat | “Be nice to yourself:   - Reward yourself for staying off cigarettes, even for one day. - Save the money you are not spending on cigarettes to buy yourself something nice.” |
| 11.1 Pharmacologic-al support | 11. Regulation | “There are treatments available that can at least double your chances of quitting…” |
| 12.1 Restructuring the physical environment | 12. Antecedents | “If you like to smoke when chatting on the phone, move the ashtray.” |

**Table S4 – BCTs utilised within** Section 2 – Physical Activity

| **BCT Label** | **BCT group** | **Example of how the BCT was used** |
| --- | --- | --- |
| 1.1 Goal setting (behaviour) | 1. Goals and planning | “Your aim is to build up to 30 minutes of moderate activity every day and reduce the amount of time you spend being inactive.”  “To help guide the intensity of your physical activity/exercise: moderate intensity activity is when you’re working hard enough to raise your heart rate and break into a sweat.” |
| 1.2 Problem solving | 1. Goals and planning | “Finding a way of being active can sometimes be hard, so here are some tips on how to make it easier for yourself:   - Choose an activity you enjoy! - Listen to music whilst you exercise. - Try and find activities that fit in with your lifestyle and that you can do on most days of the week.”   “Give the car a rest – walk or cycle to the shops, church or work.” |
| 2.3 Self-monitoring of behaviour | 2. Feedback and monitoring | “A general target to aim for is about 7,500 steps/day although some people will manage more than this…”  “You will be able to talk but unable to sing the words to a song (the ‘talk-sing’ test).”  “Physical activity and exercise record”.  “Please continue writing at the end of the manual, use a separate diary or an app on your phone to record your activity levels.” |
| 3.1 Social support (unspecified) | 3. Social support | “….set your own personal target after talking to the facilitator.”  “Sexual counselling”. |
| 3.2 Social support (practical) | 3. Social support | “Chest Heart and Stroke (NI) organise exercise classes (called Post Rehab Exercise Programme – PREP) for patients and can be accessed directly through the Association.” |
| 3.3 Social support (emotional) | 3. Social support | “Having company can make exercising more enjoyable.”  “Discuss the problem with your partner so that they know that they aren’t putting you off.” |
| 4.1 Instruction on how to perform the behaviour | 4. Shaping knowledge | Written explanation of 5 exercises to try at home. |
| 6.1 Demonstration of the behaviour | 6. Comparison of behaviour | The home exercise programme is illustrated to the reader through appropriate diagrams. |
| 7.1 Prompts/cues | 7. Associations | For being physically active:  “- Keep your walking shoes near the door.  - Keep your golf clubs or swim suit in the boot of the car.” |
| 8.1 Behavioural practice/ rehearsal | 8. Repetition and substitution | “Add activity to your daily routine – begin with three 10-minute walks spread throughout your day, for example.” |
| 9.1 Credible source | 9. Comparison of outcomes | “Some GPs can also refer you to your local council gyms through local initiatives, e.g. Healthwise scheme.” |
| 11.2 Reduce negative emotions | 11. Regulation | “This often leads to avoiding sex or not enjoying it when it does happen. In fact, it seems to be very rare for a person to have a TIA or stroke after having sex.” |
| 12.5 Adding objects to the environment | 12. Antecedents | “A pedometer…” |
| 13.2 Framing/ reframing | 13. Identity | “It is important to realise that sex is no different from any other kind of exercise. It does not put a special kind of strain on your brain.” |

**Table S5 – BCTs utilised within** Section 3 – Healthy Eating and Alcohol

| **BCT Label** | **BCT group** | **Example of how the BCT was used** |
| --- | --- | --- |
| 1.1 Goal setting (behaviours) | 1. Goals and planning | “Safe limits of alcohol intake:   1. 14 units per week for both men and women; 2. spread your alcohol intake evenly throughout the week; 3. maximum of 2-3 units per day.”  - “ I will use only semi-skimmed milk. - I won’t have second helping. - I will eat a healthy breakfast every morning like Weetabix or porridge….” |
| 1.3 Goal setting (outcome) | 1. Goals and planning | “Aim to lose weight slowly. Short-term or quick-fix diets are not good – you need to make changes you can keep to long-term.” |
| 1.4 Action planning | 1. Goal and planning | “Recommendations to adopt a Mediterranean-style diet:….”  “Make out a shopiing list with all the healthy foods you plan to buy, then take it with you to the shop.” |
| 1.5 Review behaviour goal(s) | 1. Goal and planning | “Keep a Food Diary….You might be surprised to see what your Food Diary looks like after one week! Try writing this down and discussing it with the facilitator.” |
| 2.3 Self-monitoring of behaviour | 2. Feedback and monitoring | “Set Targets for Yourself…” |
| 4.1 Instruction on how to perform the behaviour | 4. Shaping knowledge | “There are four main messages when eating for a healthy brain and heart:   - Eat less fat – especially saturated fat. - Eat more fibre – fruits, vegetables, cereals. - Eat less salt. - Eat less sugar.”   “Here are some helpful tips to make it easier for you to switch to healthier eating choices – and to enjoy them!” |
| 5.1 Information about health consequences | 5. Natural consequences | “A healthy Mediterranean diet can help prevent further brain disease, as well as other diseases such as heart attacks, by:   1. Lowering cholesterol in the blood vessels around your heart. 2. Controlling your weight, which affects your blood pressure.   Supplying you with vitamins and antioxidants, which help to keep your blood vessels in good shape.” |
| 5.6 Information about emotional consequences | 5. Natural consequences | “After a few weeks you may find that you can enjoy yourself just as much on a lot less alcohol.” |
| 6.2 Social comparison | 6. Comparison of behaviour | “A Patient’s Story”  A patient details their experience of suffering a TIA. |
| 8.1 Behavioural practice/ rehearsal | 8. Repetition and substitution | “Eat more fruit and vegetables – aim for minimum 5 portions/day….oily fish (2/3 times/week).” |
| 8.2 Behaviour substitution | 8. Repetition and substitution | “Switch to olive oil and rapeseed oil instead of lard or other vegetable oils   - Switch to olive oil spreads instead of butter or margarine”.   “….try making every other drink a low-alcohol drink.”  “Try half-fat or low-fat dairy products….” |
| 9.1 Credible source | 9. Comparison of outcomes | “A Patient’s Story”  A patient details their experience of suffering a TIA. |
| 10.3 Non-specific reward | 10. Reward and threat | “Enjoy a treat once a week as a reward.” |

**Table S6 – BCTs utilised within** Section 4 – Stress and Fatigue

| **BCT Label** | **BCT group** | **Example of how the BCT was used** |
| --- | --- | --- |
| 1.1 Goal setting (behaviour) | Goals and planning | “- Keep active – a healthy body helps keep a healthy mind.”  “…take adequate rest periods during the day….” |
| 1.2 Problem solving | 1. Goals and planning | “The way we think about a situation is part of what makes it stressful. It’s not always the situation itself that matters most, but our response to it.”  “Practical Ways to Help you Control your Stress” |
| 2.3 Self-monitoring of behaviour | 2. Feedback and monitoring | “…keeping a diary of your activities can help with this.” |
| 3.1 Social support (unspecified) | 3. Social support | “If you are finding it difficult to sleep, discuss this with your GP or another health professional.” |
| 3.2 Social support (practical) | 3. Social support | “Speak with your GP or healthcare professional who can suggest some treatments, e.g. ….antidepressant medication” |
| 3.3 Social support (emotional) | 3. Social support | “Speak with your GP or healthcare professional who can suggest some treatments, e.g. counselling…” |
| 4.2 Information about Antecedents | 4. Shaping knowledge | “two people might experience the same stressful situation in two different ways:  One might say it makes them feel helpless against an impossible barrier, as in a nightmare or a trap, with pressure from both sides – like being the meat in a sandwich.  Another person might view the same situation as an obstacle which can be overcome – an exciting opportunity or a challenge, like a successful juggler.” |
| 5.1 Information about health consequences | 5. Natural consequences | “Long periods of stress can lead to:   - High blood pressure - Muscle tension and backache…” |
| 5.3 Information about social and environmental consequences | 5. Natural consequences | “Without stress and adrenaline, we might never get anything done!” |
| 5.6 Information about emotional consequences | 5. Natural consequences | “Long periods of stress can lead to:  - Frustration, irritability and anxiety…” |
| 9.1 Credible source | 9. Comparison of outcomes | “..speak to your GP…” |
| 11.2 Reduce negative emotions | 11. Regulation | “Try not to be a perfectionist in everything.” |

**Table S7 – BCTs utilised within** Section 5 - Medication

| **BCT Label** | **BCT group** | **Example of how the BCT was used** |
| --- | --- | --- |
| 1.2 Problem solving | 1. Goals and planning | “You may find it useful to make a list of all the medicine you have to take, at which time and connect it with something you usually do at that time – like your morning wash, eating lunch, getting changed after work or going to bed.” |
| 1.4 Action planning | 1. Goals and planning | “You may find it useful to make a list of all the medicine you have to take, at which time and connect it with something you usually do at that time – like your morning wash, eating lunch, getting changed after work or going to bed.” |
| 7.1 Prompts/cues | 7. Associations | “You may find it useful to make a list of all the medicine you have to take, at which time and connect it with something you usually do at that time – like your morning wash, eating lunch, getting changed after work or going to bed.” |
| 8.1 Behavioural practice/ rehearsal | 8. Repetition and substitution | “If you always take them at these times it will soon become a habit and you are less likely to forget.” |
| 8.3 Habit formation | 8. Repetition and substitution | “If you always take them at these times it will soon become a habit and you are less likely to forget.” |
| 9.1 Credible source | 9. Comparison of outcomes | “…ask your doctor.” |
| 11.1 Pharmacologic-al support | 11. Regulation | “Medication to Help your Brain”. |

**Table S8 – BCTs utilised within Section 6 – Community Support**

| **BCT Label** | **BCT group** | **Example of how the BCT was used** |
| --- | --- | --- |
| 3.2 Social support (practical) | 3. Social support | “A friend or family member may join you, for example, in starting an exercise programme or indeed you may quit smoking together.” |
| 9.1 Credible source | 9. Comparison of outcomes | “Your facilitator, doctor or practice nurse will help you to identify organisations and services in your area that can help you in whatever positive lifestyle changes you choose to make.” |
